# Pathological Mechanisms Linking Diabetes Mellitus and Alzheimer’s Disease: the Receptor for Advanced Glycation End Products (RAGE)

**DOI:** 10.3389/fnagi.2020.00217

**Published:** 2020-07-22

**Authors:** Yanyan Kong, Fushuai Wang, Jiao Wang, Cuiping Liu, Yinping Zhou, Zhengqin Xu, Chencheng Zhang, Bomin Sun, Yihui Guan

**Affiliations:** ^1^Department of Neurosurgery, Ruijin Hospital, Shanghai Jiao Tong University School of Medicine, Shanghai, China; ^2^PET Center, Huashan Hospital, Fudan University, Shanghai, China; ^3^Laboratory of Molecular Neural Biology, School of Life Sciences, Shanghai University, Shanghai, China

**Keywords:** advanced glycation end products, RAGE, Alzheimer’s disease, diabetes, PI3K

## Abstract

Diabetes and Alzheimer’s disease (AD) place a significant burden on health care systems in the world and its aging populations. These diseases have long been regarded as separate entities; however, advanced glycation end products (AGEs) and the receptors for AGEs (RAGE) may be a link between diabetes and AD. In our study, mice injected with AGEs through stereotaxic surgery showed significant AD-like features: behavior showed decreased memory; immunofluorescence showed increased phosphorylated tau and APP. These results suggest links between diabetes and AD. Patients with diabetes are at a higher risk of developing AD, and the possible underlying molecular components of this association are now beginning to emerge.

## Introduction

Accumulated evidence suggests that sporadic cases account for more than 95% of the total cases of Alzheimer’s disease (AD; Levin, 2019). AD is a neurodegenerative disease with progressive memory cognitive dysfunction as the main clinical manifestation. The hallmark lesions observed in the brain of patients with AD result from the formation of numerous neurofibrillary tangles (NFTs) and senile plaques (SPs) composed of hyperphosphorylated tau and Aβ, respectively (Lane et al., 2018). Studies have implicated diabetes mellitus (DM) as a strong risk factor for the development of AD (Jayaraj et al., 2020). DM shares pathological features with AD, such as impaired insulin signaling, increased oxidative stress, increased amyloid-beta (Aβ) production, tauopathy, and cerebrovascular complications (Shinohara and Sato, 2017), and therefore appears to be closely related to AD.

Glycosylation is an important non-enzymatic reaction between reducing sugars and amines (Singh et al., 2018). Lysine or arginine residues of proteins and glucose, moving towards the Schiff-base, convert themselves to the more stable aminomethyl ketone by the Amadori rearrangement and finally result in the formation of advanced glycation end (AGE) products (Bunn and Higgins, 1981; Hartog et al., 2007; Rabbani and Ahn, 2019). The synthesis of AGEs is irreversible, and although the process of cell death is slow and insignificant, the non-enzymatic saccharification process accompanied by neuron metabolism seriously affects the normal functioning of the nervous system (Kamynina et al., 2018). Therefore, AGEs and the receptor for advanced glycation end-products (RAGE) may play an important role in disease pathogenesis. Many studies have shown that the accumulation of AGEs is a major factor in the incidence and development of several diabetic complications and neuropathies (Hammes et al., 1999; Negre-Salvayre et al., 2009; Singh et al., 2014; Nowotny et al., 2015; Yamagishi et al., 2015; Hashimoto et al., 2016; Kumar Pasupulati et al., 2016), and the accumulation of AGEs under *in vivo* conditions is associated with secondary complications related to diabetes in hyperglycemic environments (Garay-Sevilla et al., 2005). Furthermore, AGEs are closely linked to amyloid-based neurodegenerative diseases (Vicente Miranda and Outeiro, 2010; Li et al., 2012; Simó et al., 2017). Salahuddin et al. (2014) showed that disrupting the AGE-RAGE interaction can effectively prevent the development of AD, and AGEs are therefore considered promising drug targets for AD. Studies also show that the formation of AGEs will pass through and interfered with H1, H2A, and H3 histones by causing structural changes. The normal functioning of serotonin affects chromatin structure and function, leading to secondary complications which in turn aggravates the diabetic condition (Ashraf et al., 2014, 2015a,b). The above evidence reveals that AGEs have a role in both diabetes and AD in humans as well as in disease models.

Studies also have found that AGEs induce oxidative stress in neurons, promoting the release of neuroinflammatory cytokines and Aβ (Yan et al., 1996; Baig et al., 2018). At the same time, extracellular AGEs can also affect neuronal function through RAGE. Studies have shown that RAGE also interacts with and mediates the cytotoxicity of Aβ (Wang et al., 2018). For example, the combination of RAGE and Aβ can activate the inflammatory signaling pathway, release ROS to produce oxidative stress, and cause neuroinflammation, cause mitochondrial and neuronal dysfunction (Deane et al., 2008), and affect the mitogen-activated protein kinase signaling pathway (Deane, 2012). RAGE also accelerates the absorption and transport of Aβ, which causes Aβ to pass through the blood-brain barrier and into the central nervous system by endocytosis (Deane et al., 2003), causing cerebrovascular dysfunction and eventually leading to neurovascular inflammation and subsequent increase in synaptic toxicity (Deane and Zlokovic, 2007), which in turn affects the normal functioning of the central nervous system (Zhang et al., 2011; Galasko et al., 2014; Wang et al., 2014; Cai et al., 2016; Fang et al., 2018). The elevated expression of RAGE activates the nuclear transcription factor NF-κB, resulting in a positive feedback effect on inflammation (Wan et al., 2015; Fang et al., 2018). Aβ activation of RAGE increases the expression of proinflammatory cytokines such as TNF-α, interleukin-6, and macrophage colony-stimulating factor (Dukic-Stefanovic et al., 2003), which accelerates the occurrence and development of AD. The RAGE signaling pathway also plays an important role in AGE-induced tau phosphorylation and spatial memory impairment. In SK-N-SH cells, primary hippocampal neurons, and rats, AGEs induce tau hyperphosphorylation *via* the RAGE/GSK-3 pathway (Li et al., 2012; Son et al., 2012). AGEs also block the BDNF-TrkB signaling pathway in rat brain and N2A cells (Li et al., 2012), activate the GSK-3β kinase at Ser9, phosphorylate GSK-3, and induce tau hyperphosphorylation (Wu et al., 2019). Also, the deposition of AGEs activates microglia and nicotinamide adenine dinucleotide phosphate (NADPH) oxidase, resulting in the release of ROS and the formation of peroxynitrite, which oxidizes proteins, lipids, and DNA (Nam et al., 2012), eventually causing neuronal death. Co-immunoprecipitation studies have found that almost all AGE-immunoreactive neurons contain phosphorylated tau protein (Qi et al., 2017), which indicates that AGEs play an important role in tau protein hyperphosphorylation. As compared to AD mice, the phosphorylation levels of tau were increased in the offspring mouse model of diabetes and AD hybridization (Pdx1^+/–^/APP/PS1); furthermore, the production of Aβ was increased and the clearance of Aβ was inhibited (Guo et al., 2016). More and more evidence shows that DM is also a causative factor of AD; therefore, AD is also called type III diabetes (Luchsinger et al., 2001; Huang et al., 2014; Ahmed et al., 2015; Sridhar et al., 2015). As the RAGE signaling pathway may be an important therapeutic target in AD, computer-aided drug design has especially emerged as an efficient means of developing candidate drugs for the treatment of AD. The above observations suggest that the signal pathways mediated by AGEs/RAGE are implicated in AD-like learning and memory impairment, trigger neuroinflammation, and promote Aβ deposition and tau hyperphosphorylation.

Immunohistochemical evidence also suggests that AGEs were co-located with NFTs and SPs, indicating that AGEs can induce AD symptoms (Takeda et al., 2011). AGEs are prooxidant factors that induce oxidative stress, causing neuronal dysfunction or death. It has been reported that GSK-3β is a potential link between diabetes and AD; the excessive activation of GSK-3β can cause hyperphosphorylation of tau (Zhang et al., 2018). LiCl is an inhibitor of GSK-3 (Kurgan et al., 2019), which has been reported to alleviate the hyperphosphorylation of tau proteins caused by AGEs to a certain extent. While AGEs may play an important role in the development of AD, the mechanism remains unclear.

In hyperglycemia and diabetes, the body produces large amounts of AGEs, which accelerate and aggravate the symptoms of diabetes and its complications, such as AD (Simó et al., 2017). AGEs are elevated in AD brains where they stimulate Aβ production and colocalize with NFTs and SPs, suggesting that AGEs play an important role in the pathogenesis of AD (Cai et al., 2016). Studies have found that diabetes can aggravate the decline of tau protein lesions and spatial learning memory in the AD model. Due to advances in clinical treatment, the lifespan of diabetic patients can be further extended, with a higher global incidence of diabetes-associated AD (Ahmed et al., 2015).

AGEs play a central role in the development of AD and link neurodegenerative disease and diabetes. The stage during which AGEs impact AD and how AGEs influence tau proteins and Aβ are still unclear. The effects of increased AGEs on spatial learning and memory, as well as the effects of early inhibition of AGEs production on the behavior and pathophysiology of the AD model, have not yet been reported. Therefore, elucidating the mechanism of AGEs is important to show the association between diabetes and AD.

In previous studies, the main focus was on the mechanism of AGE-RAGE signaling in neurodegenerative diseases (Juranek et al., 2015; Batkulwar et al., 2018). The purpose of our research is to study the common target (RAGE) between diabetes and AD, and then establish the links between diabetes and AD to RAGE, and further carry out the related mechanism research. We not only studied the mechanism of RAGE in AD, but it’s also more important to link diabetes to AD through RAGE.

To explore the role of AGEs in AD and diabetes, we injected AGEs in mice and found that they showed obvious symptoms of AD; behavioral studies showed that memory was impaired, immunofluorescence and western blot showed increased APP and p-Tau levels, and PET and qPCR confirmed this increase in Aβ and Tau levels. Immunofluorescence revealed elevated RAGE, Tau, and APP levels in ZDF rats as well. Therefore, through these experiments, we demonstrated that AGEs-RAGE may be a potential link between AD and diabetes.

## Materials and Methods

### Animals

C57BL/6 mice and ZDF rats were housed in specific pathogen-free (SPF) rooms with a 12-h light-dark cycle and sufficient water and food. All animal handling protocols were approved by the Animal Ethics Committee of Fudan University (No. 20171732A680).

### Morris Water Maze

Three days before the experiment, we placed the mouse into the behavioral room to adapt to the environment. A water tank measuring 122 cm in diameter was placed into the water labyrinth and instrument collection area. The tank was filled with water and bleaching powder to make the water turbid. The camera software that measured water maze behavior was adjusted for clarity, light, and brightness using a graphic mark placed approximately 0.1 m from the bottom of the tank. On the first day, a platform was placed above the surface in a single position in the water tank. The mouse was released into the water from the east, west, south, and north directions, and the time until the mouse found the underwater platform and climbed onto it was recorded. On days 2–5, the platform was placed 1 cm below the level of the liquid. Similar to day 1, the mouse was placed in the water from four different orientations. The time until the mouse found the platform hidden under the water and climbed onto it was recorded. If the mouse did not locate the platform within 60 s, the time was recorded as 60 s. On day 6, the platform was removed and the mice were allowed to move freely in the tank for 10 min. The time spent in each quadrant was determined, with a focus on the time the mouse was in the quadrant where the platform was previously located, each quadrant was marked by a different shape on the wall.

### Y-Maze

The mice were placed in a behavioral room to adapt to the environment 3 days before the experiment. Different geometric figures were attached to the three arms of the Y-maze as visual markers. The three arms of each Y-maze were randomly divided into new arms, starting arms, and other arms. The Y-maze device was placed in the behavioral camera capture area to adjust for camera clarity, light brightness, and to set up the software. Blocking the new arm, the starting arm and the other arm were opened, allowing the mice to explore for 5 min. Subsequently, all three arms were opened and the mice were allowed to explore freely for 5 min; the time the mice spent exploring the new arm was recorded.

### Open Field

The mice were placed in a behavioral room to adapt to the environment 3 days before the experiment. A 120-cm long and wide-field camera equipment was set up in the behavioral acquisition area to adjust the camera sharpness, brightness, and set up the software. The mice were placed in the open field and allowed to move freely for 10 min. The time the mice spent in the central and surrounding areas was recorded.

### Protein Extraction

The mice were anesthetized with 2% sodium pentobarbital, and the mouse brain was quickly excised and placed in ice-cold PBS. The mouse hippocampus was isolated in frozen PBS. The hippocampus was placed in a tissue lysis buffer containing protease inhibitors. The tissue was lysed by thorough grinding on ice, was kept on ice for 2 h, and then 500 μg of the hippocampal slurry was centrifuged at 4°C for 10 min. The supernatant was transferred to a new sterile 1.5-ml Eppendorf tube.

The sample was centrifuged again at 13,000 *g* for 20 min under the same conditions. The supernatant was aspirated into a new sterilized EP tube. Each sample received 5× SDS buffer mixed with β-mercaptoethanol and was heated at 99°C for 15 min, immediately followed by a 3 min incubation on ice. The samples were then centrifuged for 3 min and used immediately or stored at −80°C until use.

### Western Blot Analysis

The percent SDS PAGE gels were determined according to the molecular weight of the target protein with a 5% separating gel. Protein ladder (1 μl) and protein samples (10 μl) were loaded onto the gel, and the gels were run at 80 V/30 min or 120 V/1.5 h. Afterward, the samples were transferred to a membrane by wet transfer. After the transfer, the membrane was blocked with 5% BSA (diluted in PBS) for 1 h, followed by incubation with the primary antibody (diluted in PBS) overnight at 4°C. The primary antibody was then aspirated off the membrane, which was washed five times with PBST for 5 min. The membrane was incubated with the fluorescent secondary antibody for 1 h at room temperature. The membrane was then washed three times with PBST for 5 min each and then scanned with the Odessey system (LI-COR, Lincoln, NE, USA).

### Immunohistochemistry

The brain tissue of the mice treated with AGEs, BSA, and AGEs+LiCl was taken from the 80°C freezer, rinsed three times with 1× PBS for 5 min each time, and incubated in PBS+0.1% TritonX-100 for 30 min at room temperature to permeate the cell membrane. The sections were then rinsed three times with 1× PBS for 5 min before adding 5% BSA-PBS for 2 h at room temperature to block non-specific binding. The blocking solution was then aspirated, and the primary antibody diluted in 1% BSA-PBS was added [APP (1:1,000, Thermo Fisher Scientific, Waltham, MA, USA), or P-TAU (1:100, Santa Cruz Biotechnology, Santa Cruz, CA, USA)] to the sections, which were incubated at 4°C overnight. The following day, the sections were washed threee times in 1× PBS for 5 min each. A fluorescent secondary antibody (1:500) diluted in 1× PBS was added in the dark and incubated for 2 h at room temperature. The secondary antibody was removed, and DAPI (1:1,000) diluted in 1× PBS was added to the sections for 5 min at room temperature. The DAPI was discarded, and the sections were washed three times in 1× PBS for 5 min each. The slides were photographed using a confocal microscope.

### RNA Extraction and Reverse Transcription and Real-Time Quantitative PCR (qPCR)

The hippocampus of the mice treated with AGEs, BSA, and AGEs + LiCl was extracted. Total RNA was extracted using Promega’s total RNA extraction kit following the manufacturer guidelines. The concentration of RNA was determined by measuring the absorbance at 260 nm, and 2 μg of RNA was used for first-strand cDNA synthesis using Takara’s reverse transcription kit. The mixture of cDNA samples and the indicated primers (Table 1) was subjected to at least three replicate qPCR amplification using YEASEN’s SYBR Green qPCR mix. Relative gene expression was calculated by comparing the CT value of the gene of interest with the CT value of the internal reference gene *GAPDH*.

**Table 1:** List of the primers used for qPCR.

Gene	Primer Sequence (5′ to 3′)
Tau	Forward Primer: TGGGGAACATTCCGTATGAGG
	Reverse Primer: CAGAAGCCATAACCCTTGGG
APP	Forward Primer: AACCGACTCCAGGATGACTATG
	Reverse Primer: TCTGGGGTTCCATGTAAAAGC
GAPDH	Forward Primer: TGGATTTGGACGCATTGGTC
	Reverse Primer: TTTGCACTGGTACGTGTTGAT

The qPCR primer sequences of the human genes (*APP*, *TAU*, *GAPDH*) required for the experiment were found in the Primer Bank, as shown in Table 1.

### Immunofluorescence

Rat brain slices were washed three times with 1× PBS for 5 min each, treated with 0.2% Triton-X100 (diluted in PBS) for 30 min, and then blocked with 5% BSA for 1 h. Next, the sections were incubated with RAGE, tau, and APP primary antibodies (diluted in PBS) at 4°C overnight. The following day, the sections were washed three times with 1× PBS for 5 min each and then incubated with secondary antibodies for 1.5 h. Finally, DAPI was incubated for 10 min. After washing with PBS, the plate was mounted with a fluorescent mounting plate. The expression of RAGE, tau, and APP was observed on confocal microscopy.

## Results

### Behavioral Experiments Reveal Abnormal Behavior in AGE-Treated Mice

**Figure 1:**
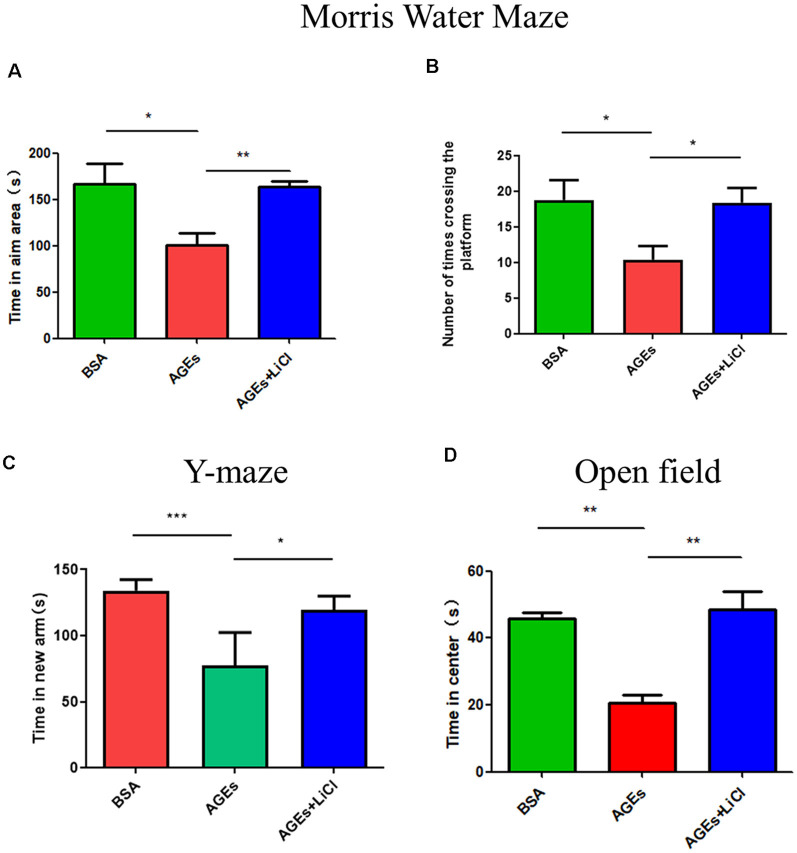
Mice injected with advanced glycation end products (AGEs) show dementia-like behavior. **(A,B)** Water maze experiment was performed on three groups of mice to observe the dementia-like behavior. **(A)** The time that the three groups of mice stayed in the target area was measured. **(B)** The number of times the three groups of mice crossed the platform. The criterion for judging the entry of the mouse into the target area was the entry point of the mouse torso center point. **(C)** Y-maze experiment measured the residence time of three groups of mice injected with BSA, AGEs, AGEs + LiCl in the brain in the new arm of the Y-maze. **(D)** Open-field experiment measured the length of stay of the three groups of mice in the central area time. For each group of mice, *n* = 5. Data are expressed as mean ± standard deviation. One-way analysis of variance (ANOVA). *p < 0.05, **p < 0.01, ***p < 0.001.

To investigate whether behavior changes develop in mice after the injection of AGEs, we studied the behavior of mice using the Morris Water Maze, Y-maze, and open field experiments. In the water maze test, compared with the control group, the AGE mice stayed in the target area for significantly lesser time, indicating that AGEs impair memory in mice. However, when treated with LiCl, the time spent in the target area significantly increased compared to that of AGE mice, indicating that LiCl can mitigate memory impairment (Figure 1A). Similarly, the number of times AGE mice crossed the platform decreased compared with that of the control group; however, when AGE mice were treated with LiCl, the number of times the mouse crossed the platform significantly increased compared to that of AGE mice (Figure 1B). This indicated that AGEs impair the memory of mice, while LiCl can alleviate the damage of AGEs.

Y-maze is an effective way to assess short-term memory in mice (Kraeuter et al., 2019). In the Y-maze experiment, compared with the control group, AGE mice stayed in the new arm for significantly lesser time. However, treatment with LiCl increased the time spent in the new arm, indicating that AGEs damage the memory capacity of mice, while LiCl can alleviate the damage of AGEs (Figure 1C). In the open field experiment, compared with the control group, AGE mice stayed in the central area for a significantly shorter time. When AGE mice were treated with LiCl, however, the time spent in the central area significantly increased. This indicated that AGEs increased the anxiety behavior of mice, while the addition of LiCl eased the anxiety behavior (Figure 1D).

### P-tau and APP Increased in the Hippocampus of AGE Mice

**Figure 2:**
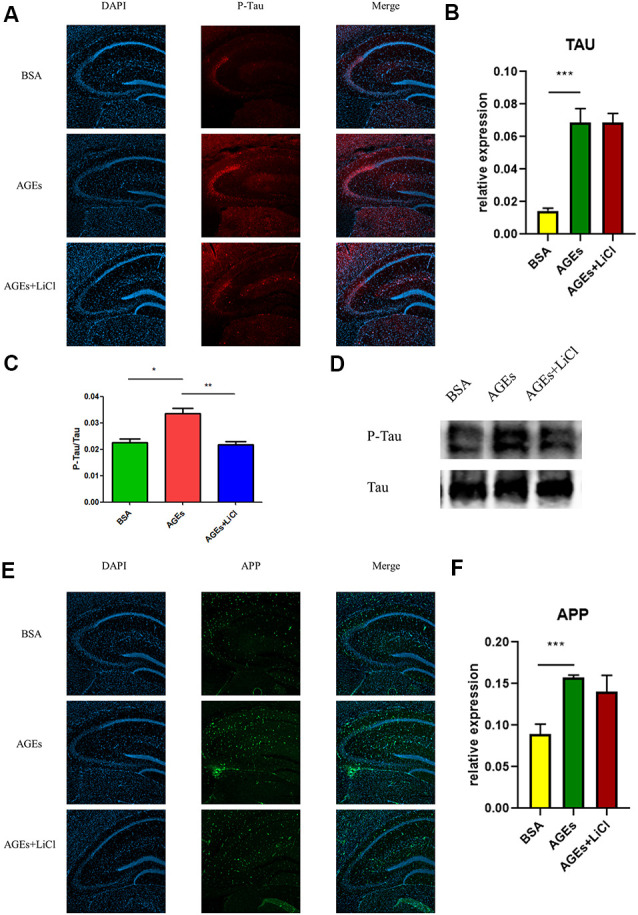
Upregulation of Alzheimer’s disease (AD)-related protein expression in advanced glycation end (AGE)-injected mice. **(A–C)** Expression of P-tau in the hippocampus of BSA, AGEs, AGEs + LiCl treated mice. **(A)** P-tau in the hippocampus of three groups of mice was stained with immunofluorescence (red), and nuclei were stained with DAPI (blue). **(B)** qPCR was performed on the hippocampus of the three groups of mice, and the expression of tau-related proteins was measured. The expression level was normalized to the average expression level in the BSA injection group; **(C,D)** Western blotting was performed on the hippocampus of the three groups of mice, and the ratio of the expression levels of P-tau to tau protein in the three groups of mice was calculated and expressed as P -tau/tau value. The gray density was normalized to the average gray density of the tau protein. **(E,F)** APP protein expression in the hippocampus of three groups of mice. **(E)** APP proteins in the hippocampus of the three groups of mice were stained with immunofluorescence (green), and the nuclei were stained with DAPI (blue). **(F)** qPCR was performed on the hippocampus of three groups of mice to measure the expression of APP-related proteins in the hippocampus. The expression level was normalized to the average expression level of the injected BSA group. For each group of mice, *n* = 3. Data are expressed as mean ± standard deviation. One-way ANOVA. *p < 0.05, **p < 0.01, ***p < 0.001.

To investigate whether mice injected with AGEs develop AD symptoms, we analyzed the expression of P-tau (Phospho-Tau-S356 Rabbit pAb) and APP in the hippocampus of the mice by immunofluorescence, Western blot, and qPCR. The results showed that compared with the BSA control group, the expression of P-tau was significantly up-regulated in AGE mice, while it was not significantly changed in AGE mice treated with LiCl (Figure 2A). Compared with the control group, the mRNA expression of tau was significantly up-regulated in AGE mice, while it was not significantly changed in AGE mice treated with LiCl (Figure 2B). The same results were verified by immunoblotting (Figures 2C,D). Compared with the BSA control group, in AGE mice, the expression of APP protein was significantly up-regulated, while it was not changed in AGE mice treated with LiCl (Figure 2E). At the mRNA level, compared with the control group, the mRNA expression of APP in the AGE mice was significantly up-regulated, with no effect of LiCl (Figure 2F). This indicated that, compared with the control, the AGE mice successfully developed AD symptoms, which were mitigated when treated with LiCl.

**Figure 3:**
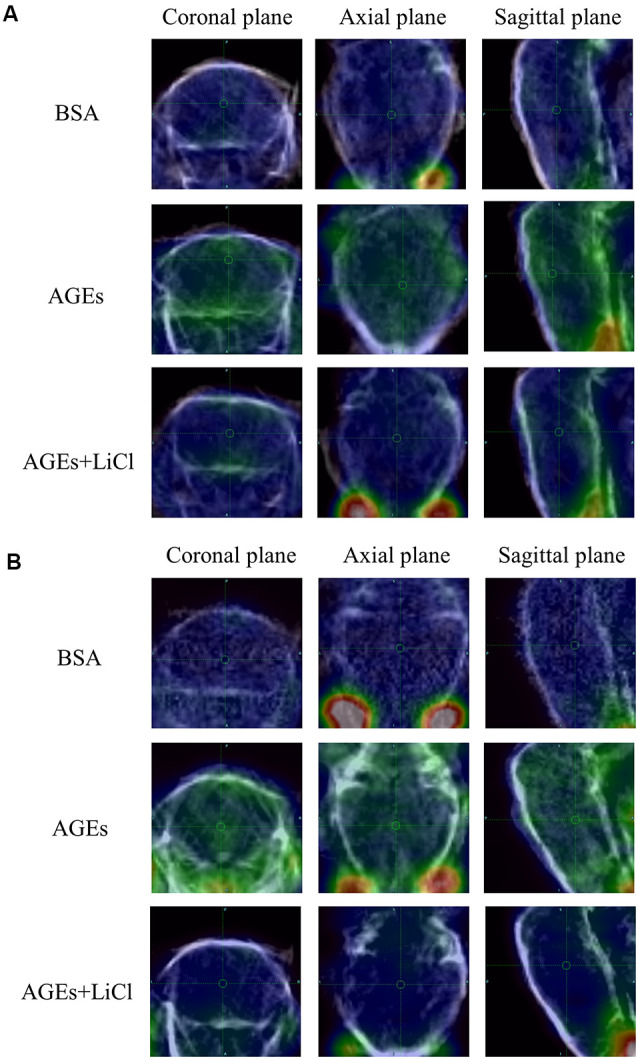
AGE mouse PET image. **(A)** Mice injected with 18F-AV45. Green represents Aβ. **(B)** Mice injected with 18F-PBB3. Green represents tau.

At the same time, the increase in tau and Aβ in living mice was further verified by PET (Figures 3A,B). AV45 and PBB3 injections, respectively, showed that tau and Aβ in AGE mice increased significantly, but were relieved when treating with LiCl. This result further demonstrates that AGEs can promote the occurrence of AD symptoms.

### AGEs Increase Tau Phosphorylation *via* the PI3K Signaling Pathway

**Figure 4:**
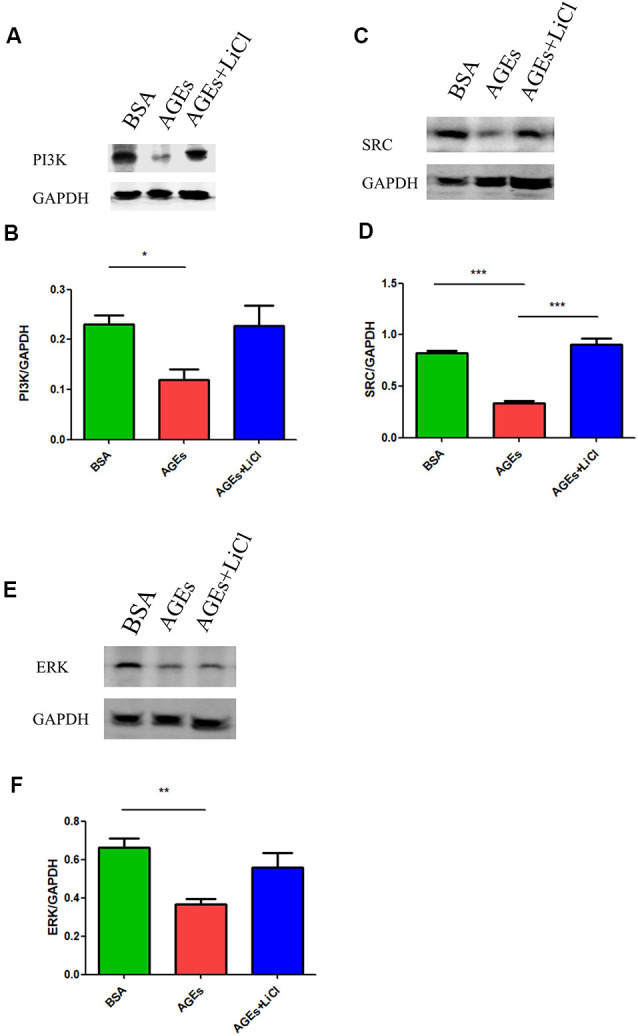
The upregulation of AGEs inhibits the PI3K/SRC/ERK signaling pathway. **(A,B)** Western blotting detection of PI3K protein in the hippocampus of three groups of mice, normalized gray density to the average gray density of GAPDH. **(C,D)** Western blotting detection of SRC protein in the hippocampus of three groups of mice. Gray density was normalized to the average gray density of GAPDH. **(E,F)** Western blotting was performed on ERK proteins in the hippocampus of the three groups of mice, and the gray density was normalized to the average gray density of GAPDH. For each group of mice, *n* = 3. Data are expressed as mean ± standard deviation. One-way ANOVA. *p < 0.05, **p < 0.01, ***p < 0.001.

Many studies have shown that increased phosphorylation of tau is closely related to the PI3K signaling pathway (Wang et al., 2018, 2019; Wei et al., 2019; Zhao et al., 2019). Activation of the PI3K signaling pathway can reduce the over-phosphorylation of tau (Xiong et al., 2020). Therefore, we speculated whether AGEs cause increased tau phosphorylation by affecting the PI3K signaling pathway. To verify our conjecture, we detected the changes in the expression of PI3K protein and its downstream SRC and ERK proteins in the hippocampus of AGE and AGE+LiCl mice. Compared with controls, AGEs significantly down-regulated the protein expression of PI3K in the hippocampus of mice. However, this was up-regulated again after treatment with LiCl, indicating that AGEs may affect the memory of mice through the PI3K signaling pathway and LiCl may alleviate the damage of AGEs to brain function to some extent (Figures 4A,B). Moreover, the protein expression of SRC in the hippocampus of AGE mice was significantly down-regulated, while it was significantly up-regulated again when treated with LiCl, indicating that the down-regulation of PI3K resulted in a decrease in SRC (Figures 4C,D). Furthermore, the expression of ERK protein in the hippocampus of AGE mice was significantly decreased. LiCl, however, had no significant effect on the expression of ERK. There was an upward trend of ERK, indicating that AGEs ultimately lead to a decrease in ERK protein through the PI3K signaling pathway, which may be the main signaling pathway leading to phosphorylation of tau protein, and the addition of LiCl can effectively inhibit the action of AGEs (Figures 4E,F).

### ZDF Rats Show Increased RAGE, APP, and Tau

**Figure 5:**
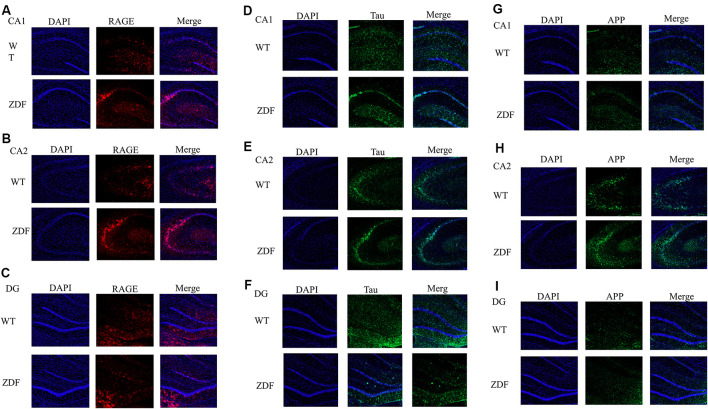
ZDF rats show AD symptoms. **(A–C)** Staining rat hippocampus with RAGE antibody (red) and the nuclei with DAPI (blue). **(D–F)** Staining rat hippocampus with tau antibody (green) and the nuclei with DAPI (blue). **(G–I)** Staining rat hippocampus with APP antibody (green) and the nuclei with DAPI (blue).

To further verify the changes of RAGE in diabetes, we investigated whether there is a link between diabetes and AD by detecting changes in APP and tau in ZDF rats. Using immunofluorescence, we found that compared with normal wild rats, RAGE expression in the CA2–CA3 region significantly increased and was aggregated (Figures 5A,B). RAGE expression in the CA1 and DG regions was similar (Figure 5C). When comparing the expression of tau protein, we found that tau expression in wild SD rats was evenly distributed in the hippocampus, while tau expression in ZDF rats showed an aggregated state (Figures 5D–F). Similarly, we found that APP showed a clear upward regulation in the hippocampus of ZDF rats (Figures 5G–I). These results indicate that RAGE expression is up-regulated in diabetic rats and that AD-like symptoms (increased tau aggregation and APP expression) occur in the hippocampus of diabetic rats, confirming that there is indeed an association between diabetes and AD.

## Discussion

This study investigated the association between diabetes and AD through the treatment of mice with AGEs. First, memory dysfunction in AGE mice was verified by behavior (Figure 1). Next, immunofluorescence, qPCR, and Western blotting confirmed an increase in P-tau and APP in the hippocampus of AGE mice (Figures 2, 3). We then speculated that AGEs may cause tau protein hyperphosphorylation through the PI3K signaling pathway (Figure 4). At the same time, LiCl improved these symptoms caused by AGEs. Finally, it was confirmed by immunofluorescence experiments in ZDF rats that diabetic rats develop AD symptoms (Figure 5). Therefore, our data illustrate the close relationship between diabetes and AD.

When diabetic patients are in a state of hyperglycemia for a long time, glucose will form covalent compound AGEs with plasma. AGEs not only form intracellular and cellular diplomatic links with proteins but also form crosslinks with other endogenous key molecules including lipids and nucleic acids, thereby promoting the development of diabetic complications (Singh et al., 2014). Cognitive dysfunction has been documented as one of the complications of diabetes, suggesting a close link between diabetes and AD (Jash et al., 2020). One of the main symptoms of AD is impaired memory (Sharma et al., 2020); therefore, to test whether the memory is altered in mice after the injection of AGEs, we conducted the Morris Water Maze, Y-maze, and open field tests and found that the memory of AGE mice decreased significantly and anxiety behavior increased, which is consistent with previous AD research (Tian et al., 2019; Du et al., 2020). Subsequently, to further study whether the brain of AGE mice can develop AD symptoms, we assessed the expression of AD-related proteins such as P-tau and APP, which were significantly increased in the hippocampus of AGE mice. This indicates that AGEs promote the occurrence of AD symptoms.

Much research has been done on the relationship between diabetes and AD. Some researchers have proposed that insulin can affect the phosphorylation of tau protein through the GSK-3 signaling pathway (Jash et al., 2020) and that LiCl is an inhibitor of GSK-3 (Kurgan et al., 2019). Therefore, when treated with LiCl, AGEs were not able to phosphorylate tau in large amounts, thereby improving AD symptoms in AGE mice. However, our results did not show that AD symptoms in AGE mice were significantly improved after treatment with LiCl; therefore, we speculate that the GSK-3 signaling pathway is only one of the links between diabetes and AD and other mechanisms have not been studied. Therefore, through AGEs, we speculate that there is a complex relationship between diabetes and AD, and its specific molecular mechanism requires further study.

A previous study reported that AGEs/RAGE interactions can stimulate the activation of multiple signaling cascades, including PI3K, and reduce the mRNA expression of downstream proteins of PI3K signaling pathways, such as AKT2 (Abdelmageed et al., 2019). These data indicate that AGEs/RAGE interactions occur through the PI3K signaling pathway. Furthermore, studies have shown that normal activation of the PI3K signaling pathway can reduce tau hyperphosphorylation (Xiong et al., 2020). Our previous experimental results showed that the proportion of phosphorylated tau protein increased in the hippocampus of mice when AGEs were injected. We further explored whether this is caused by the PI3K signaling pathway. We found that when mice were injected with AGEs, the expression of PI3K, SRC, and ERK proteins in the hippocampus were decreased, and the expression of PI3K and SRC increased again after injection of its inhibitor LiCl (Figure 3). The class III PI3K/Beclin-1 pathway has certain autophagic activity to promote autophagy clearance of tau (Chen et al., 2020), suggesting that blocking of PI3K-related pathways will hinder tau clearance. AGEs can induce tau hyperphosphorylation in SK-N-SH cells, primary hippocampal neurons, and in SD rats through the RAGE/GSK-3 pathway (Li et al., 2012; Son et al., 2012). The deposits of AGEs will activate microglia and NADPH oxidase, affecting PI3K-related pathways, leading to ROS synthesis and release, and further leading to oxidative stress (Nam et al., 2012; Zhou et al., 2020). In turn, this leads to the deposition of tau, suggesting that blocking the PI3K-related pathways will result in tau deposition. We speculate that AGEs promote the increase in tau phosphorylation by blocking the PI3K signaling pathway and down-regulating the expression of its downstream proteins, while LiCl has a certain relieving effect. However, its specific mechanism remains to be further studied.

Finally, we performed experiments on a diabetic rat model to verify whether symptoms similar to AD would occur in the diabetic model. We found that RAGE expression in the hippocampus of ZDF rats was increased significantly. At the same time, tau and APP expression were also increased, which is similar to the phenotype observed in the AD mouse model (Gallardo and Holtzman, 2019), further illustrating the relationship between diabetes and AD.

In conclusion, mice injected with AGEs showed significant AD-like features including decreased memory and increased phosphorylated tau and APP expression. These results suggest links between diabetes and AD. Patients with diabetes are at a higher risk of developing AD, and the possible underlying molecular components of this association are now beginning to emerge.

## Data Availability Statement

All datasets presented in this study are included in the article.

## Ethics Statement

The animal study was reviewed and approved by Animal Ethics Committee of Fudan University.

## Author Contributions

YK and CZ designed the experiments. YK, FW, CL, YZ, and ZX conducted the experiments. BS and YG obtained funding and revised the manuscript. All authors read and approved the final manuscript.

## Conflict of Interest

The authors declare that the research was conducted in the absence of any commercial or financial relationships that could be construed as a potential conflict of interest.

## References

[B1] Abdelmageed M. E. Shehatou G. S. Abdelsalam R. A. Suddek G. M. Salem H. A. (2019). Cinnamaldehyde ameliorates STZ-induced rat diabetes through modulation of IRS1/PI3K/AKT2 pathway and AGEs/RAGE interaction. Naunyn Schmiedebergs Arch. Pharmacol. 392, 243–258. 10.1007/s00210-018-1583-4 30460386

[B2] Ahmed S. Mahmood Z. Zahid S. (2015). Linking insulin with Alzheimer’s disease: emergence as type III diabetes. Neurol. Sci. 36, 1763–1769. 10.1007/s10072-015-2352-5 26248483

[B4] Ashraf J. M. Ahmad S. Rabbani G. Hasan Q. Jan A. T. Lee E. J.. (2015a). 3-Deoxyglucosone: a potential glycating agent accountable for structural alteration in H3 histone protein through generation of different AGEs. PLoS One 10:e0116804. 10.1371/journal.pone.0116804 25689368 PMC4331494

[B6] Ashraf J. M. Rabbani G. Ahmad S. Hasan Q. Khan R. H. Alam K.. (2015b). Glycation of H1 histone by 3-deoxyglucosone: effects on protein structure and generation of different advanced glycation end products. PLoS One 10:e0130630. 10.1371/journal.pone.0130630 26121680 PMC4487796

[B5] Ashraf J. M. Ahmad S. Rabbani G. Jan A. T. Lee E. J. Khan R. H.. (2014). Physicochemical analysis of structural alteration and advanced glycation end products generation during glycation of H2A histone by 3-deoxyglucosone. IUBMB Life 66, 686–693. 10.1002/iub.1318 25380060

[B7] Baig M. H. Ahmad K. Rabbani G. Choi I. (2018). Use of peptides for the management of Alzheimer’s disease: diagnosis and inhibition. Front. Aging Neurosci. 10:21. 10.3389/fnagi.2018.00021 29467644 PMC5808296

[B8] Batkulwar K. Godbole R. Banarjee R. Kassaar O. Williams R. J. Kulkarni M. J. (2018). Advanced glycation end products modulate amyloidogenic APP processing and tau phosphorylation: a mechanistic link between glycation and the development of Alzheimer’s disease. ACS Chem. Neurosci. 9, 988–1000. 10.3389/fnagi.2018.00021 29384651

[B9] Bunn H. F. Higgins P. J. (1981). Reaction of monosaccharides with proteins: possible evolutionary significance. Science 213, 222–224. 10.1126/science.12192669 12192669

[B10] Cai Z. Liu N. Wang C. Qin B. Zhou Y. Xiao M.. (2016). Role of RAGE in Alzheimer’s disease. Cell. Mol. Neurobiol. 36, 483–495. 10.1007/s10571-015-0233-3 26175217 PMC11482350

[B11] Chen Y. Chen Y. Liang Y. Chen H. Ji X. Huang M. (2020). Berberine mitigates cognitive decline in an Alzheimer’s disease mouse model by targeting both tau hyperphosphorylation and autophagic clearance. Biomed. Pharmacother. 121:109670. 10.1016/j.biopha.2019.109670 31810131

[B15] Deane R. J. (2012). Is RAGE still a therapeutic target for Alzheimer’s disease? Future Med. Chem. 4, 915–925. 10.4155/fmc.12.51 22571615 PMC4973574

[B13] Deane R. Du Yan S. Submamaryan R. K. LaRue B. Jovanovic S. Hogg E.. (2003). RAGE mediates amyloid-β peptide transport across the blood-brain barrier and accumulation in brain. Nat. Med. 9, 907–913. 10.1038/nm890 12808450

[B14] Deane R. Sagare A. Zlokovic B. V. (2008). The role of the cell surface LRP and soluble LRP in blood-brain barrier Aβ clearance in Alzheimer’s disease. Curr. Pharm. Des. 14, 1601–1605. 10.2174/138161208784705487 18673201 PMC2895311

[B12] Deane R. Zlokovic B. V. (2007). Role of the blood-brain barrier in the pathogenesis of Alzheimer’s disease. Curr. Alzheimer Res. 4, 191–197. 10.2174/156720507780362245 17430246

[B16] Du Y. Liu X. Zhu X. Liu Y. Wang X. Wu X. (2020). Activating transcription factor 6 reduces Aβ1–42 and restores memory in Alzheimer’s disease model mice. Int. J. Neurosci. [Epub ahead of print]. 10.1080/00207454.2020.1715977 31928492

[B17] Dukic-Stefanovic S. Gasic-Milenkovic J. Deuther-Conrad W. Munch G. (2003). Signal transduction pathways in mouse microglia N-11 cells activated by advanced glycation endproducts (AGEs). J. Neurochem. 87, 44–55. 10.1046/j.1471-4159.2003.01988.x 12969251

[B18] Fang F. Yu Q. Arancio O. Chen D. Gore S. S. Yan S. S.. (2018). RAGE mediates Aβ accumulation in a mouse model of Alzheimer’s disease *via* modulation of β- and γ-secretase activity. Hum. Mol. Genet. 27, 1002–1014. 10.1093/hmg/ddy017 29329433 PMC6075512

[B19] Galasko D. Bell J. Mancuso J. Y. Kupiec J. W. Sabbagh M. N. van Dyck C.. (2014). Clinical trial of an inhibitor of RAGE-Aβ interactions in Alzheimer disease. Neurology 82, 1536–1542. 10.1212/WNL.0000000000000364 24696507 PMC4011464

[B20] Gallardo G. Holtzman D. M. (2019). Amyloid-β and tau at the crossroads of Alzheimer’s disease. Adv. Exp. Med. Biol. 1184, 187–203. 10.1007/978-981-32-9358-8_16 32096039

[B21] Garay-Sevilla M. E. Regalado J. C. Malacara J. M. Nava L. E. Wrobel-Zasada K. Castro-Rivas A.. (2005). Advanced glycosylation end products in skin, serum, saliva and urine and its association with complications of patients with type 2 diabetes mellitus. J. Endocrinol. Invest. 28, 223–230. 10.1007/BF03345377 15952406

[B22] Guo C. Zhang S. Li J. Y. Ding C. Yang Z. H. Chai R.. (2016). Chronic hyperglycemia induced *via* the heterozygous knockout of Pdx1 worsens neuropathological lesion in an Alzheimer mouse model. Sci. Rep. 6:29396. 10.1038/srep29396 27406855 PMC4942607

[B23] Hammes H. P. Alt A. Niwa T. Clausen J. T. Bretzel R. G. Brownlee M.. (1999). Differential accumulation of advanced glycation end products in the course of diabetic retinopathy. Diabetologia 42, 728–736. 10.1007/s001250051221 10382593

[B24] Hartog J. W. Voors A. A. Bakker S. J. Smit A. J. van Veldhuisen D. J. (2007). Advanced glycation end-products (AGEs) and heart failure: pathophysiology and clinical implications. Eur. J. Heart Fail. 9, 1146–1155. 10.1016/j.ejheart.2007.09.009 18023248

[B25] Hashimoto K. Kunikata H. Yasuda M. Ito A. Aizawa N. Sawada S.. (2016). The relationship between advanced glycation end products and ocular circulation in type 2 diabetes. J. Diabetes Complications 30, 1371–1377. 10.1016/j.jdiacomp.2016.04.024 27209548

[B26] Huang C. C. Chung C. M. Leu H. B. Lin L. Y. Chiu C. C. Hsu C. Y.. (2014). Diabetes mellitus and the risk of Alzheimer’s disease: a nationwide population-based study. PLoS One 9:e87095. 10.1371/journal.pone.0087095 24489845 PMC3906115

[B27] Jash K. Gondaliya P. Kirave P. Kulkarni B. Sunkaria A. Kalia K. (2020). Cognitive dysfunction: a growing link between diabetes and Alzheimer’s disease. Drug Dev. Res. 81, 144–164. 10.1002/ddr.21579 31820484

[B28] Jayaraj R. L. Azimullah S. Beiram R. (2020). Diabetes as a risk factor for Alzheimer’s disease in the Middle East and its shared pathological mediators. Saudi J. Biol. Sci. 27, 736–750. 10.1016/j.sjbs.2019.12.028 32210695 PMC6997863

[B29] Juranek J. Ray R. Banach M. Rai V. (2015). Receptor for advanced glycation end-products in neurodegenerative diseases. Rev. Neurosci. 26, 691–698. 10.1515/revneuro-2015-0003 26226128

[B30] Kamynina A. V. Esteras N. Koroev D. O. Bobkova N. V. Balasanyants S. M. Simonyan R. A.. (2018). Synthetic fragments of receptor for advanced glycation end products bind β-amyloid 1–40 and protect primary brain cells from β-amyloid toxicity. Front. Neurosci. 12:681. 10.3389/fnins.2018.00681 30319347 PMC6170785

[B31] Kraeuter A. K. Guest P. C. Sarnyai Z. (2019). The Y-Maze for assessment of spatial working and reference memory in mice. Methods Mol. Biol. 1916, 105–111. 10.1007/978-1-4939-8994-2_10 30535688

[B32] Kumar Pasupulati A. Chitra P. S. Reddy G. B. (2016). Advanced glycation end products mediated cellular and molecular events in the pathology of diabetic nephropathy. Biomol. Concepts 7, 293–309. 10.1515/bmc-2016-0021 27816946

[B33] Kurgan N. Whitley K. C. Maddalena L. A. Moradi F. Stoikos J. Hamstra S. I.. (2019). A low-therapeutic dose of lithium inhibits GSK3 and enhances myoblast fusion in C2C12 cells. Cells 8:1340. 10.3390/cells8111340 31671858 PMC6912290

[B34] Lane C. A. Hardy J. Schott J. M. (2018). Alzheimer’s disease. Eur. J. Neurol. 25, 59–70. 10.1111/ene.13439 28872215

[B35] Levin J. (2019). Parkinsonism in genetic and sporadic Alzheimer’s disease. Int. Rev. Neurobiol. 149, 237–247. 10.1016/bs.irn.2019.10.005 31779814

[B36] Li J. Liu D. Sun L. Lu Y. Zhang Z. (2012). Advanced glycation end products and neurodegenerative diseases: mechanisms and perspective. J. Neurol. Sci. 317, 1–5. 10.1016/j.jns.2012.02.018 22410257

[B38] Li X.-H. Lv B.-L. Xie J.-Z. Liu J. Zhou X.-W. Wang J.-Z. (2012). AGEs induce Alzheimer-like tau pathology and memory deficit *via* RAGE-mediated GSK-3 activation. Neurobiol. Aging 33, 1400–1410. 10.1016/j.neurobiolaging.2011.02.003 21450369

[B39] Luchsinger J. A. Tang M. X. Stern Y. Shea S. Mayeux R. (2001). Diabetes mellitus and risk of Alzheimer’s disease and dementia with stroke in a multiethnic cohort. Am. J. Epidemiol. 154, 635–641. 10.1093/aje/154.7.635 11581097

[B41] Nam J. H. Park K. W. Park E. S. Lee Y. B. Lee H. G. Baik H. H.. (2012). Interleukin-13/-4-induced oxidative stress contributes to death of hippocampal neurons in aβ1–42-treated hippocampus *in vivo*. Antioxid. Redox Signal. 16, 1369–1383. 22248368 10.1089/ars.2011.4175

[B42] Negre-Salvayre A. Salvayre R. Auge N. Pamplona R. Portero-Otin M. (2009). Hyperglycemia and glycation in diabetic complications. Antioxid. Redox Signal. 11, 3071–3109. 10.1089/ars.2009.2484 19489690

[B43] Nowotny K. Jung T. Hohn A. Weber D. Grune T. (2015). Advanced glycation end products and oxidative stress in type 2 diabetes mellitus. Biomolecules 5, 194–222. 10.3390/biom5010194 25786107 PMC4384119

[B44] Qi L. Chen Z. Wang Y. Liu X. Liu X. Ke L.. (2017). Subcutaneous liraglutide ameliorates methylglyoxal-induced Alzheimer-like tau pathology and cognitive impairment by modulating tau hyperphosphorylation and glycogen synthase kinase-3β. Am. J. Transl. Res. 9, 247–260. 28337257 PMC5340664

[B45] Rabbani G. Ahn S. N. (2019). Structure, enzymatic activities, glycation and therapeutic potential of human serum albumin: a natural cargo. Int. J. Biol. Macromol. 123, 979–990. 10.1016/j.ijbiomac.2018.11.053 30439428

[B46] Salahuddin P. Rabbani G. Khan R. H. (2014). The role of advanced glycation end products in various types of neurodegenerative disease: a therapeutic approach. Cell. Mol. Biol. Lett. 19, 407–437. 10.2478/s11658-014-0205-5 25141979 PMC6275793

[B47] Sharma P. Sharma A. Fayaz F. Wakode S. Pottoo F. H. (2020). Biological signatures of Alzheimer disease. Curr. Top. Med. Chem. 20, 770–781. 10.2174/1568026620666200228095553 32108008

[B48] Shinohara M. Sato N. (2017). Bidirectional interactions between diabetes and Alzheimer’s disease. Neurochem. Int. 108, 296–302. 10.1016/j.neuint.2017.04.020 28551028

[B49] Simó R. Ciudin A. Simo-Servat O. Hernandez C. (2017). Cognitive impairment and dementia: a new emerging complication of type 2 diabetes-The diabetologist’s perspective. Acta Diabetol. 54, 417–424. 10.1007/s00592-017-0970-5 28210868

[B50] Singh V. P. Bali A. Singh N. Jaggi A. S. (2014). Advanced glycation end products and diabetic complications. Korean J. Physiol. Pharmacol. 18, 1–14. 10.4196/kjpp.2014.18.1.1 24634591 PMC3951818

[B51] Singh Y. Wang T. Geringer S. A. Stine K. J. Demchenko A. V. (2018). Regenerative glycosylation. J. Org. Chem. 83, 374–381. 10.1021/acs.joc.7b02768 29227649 PMC5971087

[B52] Son S. M. Jung E. S. Shin H. J. Byun J. Mook-Jung I. (2012). Aβ-induced formation of autophagosomes is mediated by RAGE-CaMKKβ-AMPK signaling. Neurobiol. Aging 33, 1006.e11–1006.e23. 10.1016/j.neurobiolaging.2011.09.039 22048125

[B54] Sridhar G. R. Lakshmi G. Nagamani G. (2015). Emerging links between type 2 diabetes and Alzheimer’s disease. World J. Diabetes 6, 744–751. 10.4239/wjd.v6.i5.744 26069723 PMC4458503

[B55] Takeda S. Sato N. Rakugi H. Morishita R. (2011). Molecular mechanisms linking diabetes mellitus and Alzheimer disease: β-amyloid peptide, insulin signaling, and neuronal function. Mol. Biosyst. 7, 1822–1827. 10.1039/c0mb00302f 21431241

[B56] Tian H. Ding N. Guo M. Wang S. Wang Z. Liu H.. (2019). Analysis of learning and memory ability in an Alzheimer’s disease mouse model using the morris water maze. J. Vis. Exp. 152:e60055. 10.3791/60055 31736488

[B57] Vicente Miranda H. Outeiro T. F. (2010). The sour side of neurodegenerative disorders: the effects of protein glycation. J. Pathol. 221, 13–25. 10.1002/path.2682 20186922

[B58] Wan W. Cao L. Liu L. Zhang C. Kalionis B. Tai X.. (2015). Aβ(1–42) oligomer-induced leakage in an *in vitro* blood-brain barrier model is associated with up-regulation of RAGE and metalloproteinases and down-regulation of tight junction scaffold proteins. J. Neurochem. 134, 382–393. 10.1111/jnc.13122 25866188

[B59] Wang H. Chen F. Du Y. F. Long Y. Reed M. N. Hu M.. (2018). Targeted inhibition of RAGE reduces amyloid-β influx across the blood-brain barrier and improves cognitive deficits in db/db mice. Neuropharmacology 131, 143–153. 10.1016/j.neuropharm.2017.12.026 29248482

[B61] Wang S. He B. Hang W. Wu N. Xia L. Wang X.. (2018). Berberine alleviates tau hyperphosphorylation and axonopathy-associated with diabetic encephalopathy *via* restoring PI3K/Akt/GSK3β pathway. J. Alzheimers Dis. 65, 1385–1400. 10.3233/jad-180497 30175975

[B60] Wang J. Li W. Zhou F. Feng R. Wang F. Zhang S.. (2019). ATP11B deficiency leads to impairment of hippocampal synaptic plasticity. J. Mol. Cell Biol. 11, 688–702. 10.1093/jmcb/mjz042 31152587 PMC7261485

[B62] Wang X. Yu S. Hu J.-P. Wang C.-Y. Wang Y. Liu H.-X.. (2014). Streptozotocin-induced diabetes increases amyloid plaque deposition in AD transgenic mice through modulating AGEs/RAGE/NF-κB pathway. Int. J. Neurosci. 124, 601–608. 10.3109/00207454.2013.866110 24228859

[B63] Wei T. Wang Y. Xu W. Liu Y. Chen H. Yu Z. (2019). KCa3.1 deficiency attenuates neuroinflammation by regulating an astrocyte phenotype switch involving the PI3K/AKT/GSK3β pathway. Neurobiol. Dis. 132:104588. 10.1016/j.nbd.2019.104588 31470105

[B64] Wu B. Wang Y. Shi C. Chen Y. Yu L. Li J.. (2019). Ribosylation-derived advanced glycation end products induce tau hyperphosphorylation through brain-derived neurotrophic factor reduction. J. Alzheimers Dis. 71, 291–305. 10.3233/jad-190158 31381511

[B65] Xiong R. Wang X.-L. Wu J.-M. Tang Y. Qiu W.-Q. Shen X.. (2020). Polyphenols isolated from lychee seed inhibit Alzheimer’s disease-associated Tau through improving insulin resistance *via* the IRS-1/PI3K/Akt/GSK-3β pathway. J. Ethnopharmacol. 251:112548. 10.1016/j.jep.2020.112548 31917277

[B66] Yamagishi S. Nakamura N. Suematsu M. Kaseda K. Matsui T. (2015). Advanced glycation end products: a molecular target for vascular complications in diabetes. Mol. Med. 21, S32–S40. 10.2119/molmed.2015.00067 26605646 PMC4661053

[B67] Yan S. D. Chen X. Fu J. Chen M. Zhu H. Roher A.. (1996). RAGE and amyloid-β peptide neurotoxicity in Alzheimer’s disease. Nature 382, 685–691. 10.1038/382685a0 8751438

[B68] Zhang Y. Huang N. Q. Yan F. Jin H. Zhou S. Y. Shi J. S.. (2018). Diabetes mellitus and Alzheimer’s disease: GSK-3β as a potential link. Behav. Brain Res. 339, 57–65. 10.1016/j.bbr.2017.11.015 29158110

[B69] Zhang Z. Liu S. Shi R. Zhao G. (2011). miR-27 promotes human gastric cancer cell metastasis by inducing epithelial-to-mesenchymal transition. Cancer Genet. 204, 486–491. 10.1016/j.cancergen.2011.07.004 22018270

[B70] Zhao Z.-Y. Zhang Y.-Q. Zhang Y.-H. Wei X.-Z. Wang H. Zhang M.. (2019). The protective underlying mechanisms of Schisandrin on SH-SY5Y cell model of Alzheimer’s disease. J. Toxicol. Environ. Health A 82, 1019–1026. 10.1080/15287394.2019.1684007 31739764

[B71] Zhou L. Song H. Zhang Y. Ren Z. Li M. Fu Q. (2020). Polyphyllin VII attenuated RANKL-induced osteoclast differentiation *via* inhibiting of TRAF6/c-Src/PI3K pathway and ROS production. BMC Musculoskelet. Disord. 21:112. 10.1186/s12891-020-3077-z 32075617 PMC7031869

